# Design and Gas Separation Performance of Imidazolium Poly(ILs) Containing Multivalent Imidazolium Fillers and Crosslinking Agents

**DOI:** 10.3390/polym13091388

**Published:** 2021-04-24

**Authors:** Kathryn E. O’Harra, Emily M. DeVriese, Erika M. Turflinger, Danielle M. Noll, Jason E. Bara

**Affiliations:** Department of Chemical & Biological Engineering, University of Alabama, Tuscaloosa, AL 35487-0203, USA; keoharra@ua.edu (K.E.O.); emdevriese@crimson.ua.edu (E.M.D.); emturflinger@crimson.ua.edu (E.M.T.); dmnoll@crimson.ua.edu (D.M.N.)

**Keywords:** gas separation, ionic liquids, imidazolium, polyelectrolytes, crosslinkers, membranes

## Abstract

This work introduces a series of vinyl-imidazolium-based polyelectrolyte composites, which were structurally modified via impregnation with multivalent imidazolium-benzene ionic liquids (ILs) or crosslinked with novel cationic crosslinkers which possess internal imidazolium cations and vinylimidazolium cations at the periphery. A set of eight [C_4_vim][Tf_2_N]-based membranes were prepared via UV-initiated free radical polymerization, including four composites containing di-, tri-, tetra-, and hexa-imidazolium benzene ILs and four crosslinked derivatives which utilized tri- and tetra- vinylimidazolium benzene crosslinking agents. Structural and functional characterizations were performed, and pure gas permeation data were collected to better understand the effects of “free” ILs dispersed in the polymeric matrix versus integrated ionic crosslinks on the transport behaviors of these thin films. These imidazolium PIL:IL composites exhibited moderately high CO_2_ permeabilities (~20–40 Barrer), a 4–7× increase relative to corresponding neat PIL, with excellent selectivities against N_2_ or CH_4._ The addition of imidazolium-benzene fillers with increased imidazolium content were shown to correspondingly enhance CO_2_ solubility (di- < tri- < tetra- < hexa-), with the [C_4_vim][Tf_2_N]: [Hexa(Im^+^)Benz ][Tf_2_N] composite showing the highest CO_2_ permeability (P_CO2_ = 38.4 Barrer), while maintaining modest selectivities (α_CO2/CH4_ = 20.2, α_CO2/N2_ = 23.6). Additionally, these metrics were similarly improved with the integration of more ionic content bonded to the polymeric matrix; increased P_CO2_ with increased wt% of the tri- and tetra-vinylimidazolium benzene crosslinking agent was observed. This study demonstrates the intriguing interactions and effects of ionic additives or crosslinkers within a PIL matrix, revealing the potential for the tuning of the properties and transport behaviors of ionic polymers using ionic liquid-inspired small molecules.

## 1. Introduction

Membrane-based gas separations have drawn significant research attention, serving as a more economic and less energy intensive alternative to conventional separation processes (i.e., cryogenic distillation, pressure swing absorption) [1,2,3]. Robust polymeric films and ionic liquid (IL)-based materials (e.g., polyelectrolytes, supported liquid membranes) are two of the primary material classes explored in leading membrane materials research, due to the stable and rigid nature of polymeric membranes and the tunable intermolecular interactions allowed by charged materials. Polyelectrolytes and ionic liquid (IL)-based systems have been investigated as membrane materials for gas separations, as well as other related applications including ion exchange or liquid filtration systems [4,5,6,7,8,9,10]. The suitability and diversity of charged composites which are based upon these ionic material classes present countless opportunities using versatile design strategies [11]. The combined interactions between ionic moieties, paired with sophisticated structural features or functional linkages, allow for innumerous derivatives with precise chain–chain interactions which dictate chain packing, entanglement, and free volume amongst other parameters which influence the solution and diffusion of gas through a membrane [12].

Many polyelectrolytes and poly(ILs) (PILs) based upon imidazolium cations have been introduced, in conjunction with the development of imidazolium ionic liquid chemistries. Imidazolium polyelectrolytes and ILs are prevalent in the literature, due to the synthetic accessibility for the incorporation of the imidazolium group, in addition to important interactions for separation processes such as its affinity for CO_2_ vs. other non-polar gases [13,14,15]. Many imidazolium polymers rely upon free-radical polymerization techniques to incorporate imidazolium groups pendant to the backbone, often utilizing vinyl- or styrenyl-imidazole monomers or linear/difunctional crosslinkers. While several approaches have been demonstrated using vinylimidazolium monomeric or polymeric precursors to produce ion gels, ion exchange membranes, or crosslinked composites geared toward understanding the trends in altered conductivity and thermal or mechanical properties, these composites using common imidazolium ILs or simple linear, alkyl-bridged difunctional crosslinking agents serve as the foundation for more sophisticated and structurally defined composite systems [16,17,18,19,20,21,22,23,24]. It should be noted that most research existing in the literature primarily focuses on the structural modification of the repeat unit of these PIL homopolymers or copolymers, including connective or pendant segments of the monomeric precursor and the cation/anion pairing, though some studies evaluate the specific effects of added IL, crosslinking agents, or salts.

**Figure 1:**
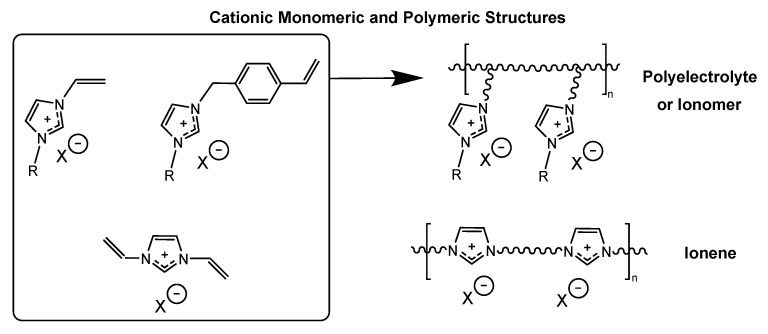
General representation of imidazolium-based building blocks for cationic polymers including polyelectrolytes and ionomers, compared with structurally similar ionenes.

Contrary to imidazolium polyelectrolytes formed conventionally via free radical polymerization with charged features being pendant to the backbone, our group has reported several works related to the gas separation performance of polyimide and polyamide imidazolium ionenes, polymers formed via the Menshutkin reaction between a bis(imidazole) and α,ω-dihalide wherein the charged group is directly incorporated within the main chain alternating with other functional and structural features along the backbone [25,26,27,28] (Figure 1). Our group has previously reviewed the synthetic pathways and applications of imidazolium ionenes, which are an extremely tunable platform which can further be modified by the integration of free IL or multivalent fillers to promote structuring within the polymeric ionene matrix [15,27,29]. These imidazolium ionene composites with IL yielded enhanced permeabilities, in comparison with their corresponding neat ionene counterparts, and high selectivities for CO_2_ relative to N_2_ and CH_4_. The addition of “free” IL into ionenes was shown to induce self-assembly and promote structuring within the ionic composites, resulting in enhanced CO_2_ permeability. Imidazolium-mediated systems have been shown to possess an affinity for and the ability to bind CO_2_, which is beneficial for the design of CO_2_-selective membranes based upon a solution-diffusion transport mechanism. More recently, we have explored the design of more sophisticated, multivalent ionic fillers as a means of altering the local ordering and structuring within an ionic polymeric matrix [27].

Nonetheless, these compositional concepts and structural fine tuning can be applied to poly(ILs) similarly to ionene–IL composites, to better understand the how these charged fillers interact with the pendant ionic groups and probe the effects on structure, homogeneity, mechanical properties, and transport behaviors. While the nature of the ionic group and the segment tethering the charged moiety to the PIL backbone are certainly influential, the tunability and versatility of PIL-IL composites is intriguing. Several reviews have addressed the potential for novel structures and limitless composite compositions employing these concepts due to the potential variation of both the PIL and “free” IL components [11,30,31,32]. While others have explored this PIL-IL composite platform, these ideas have not moved past simple, structurally similar compositions produced from well-known monomers and common ILs toward more advanced networks with hierarchical structuring.

The introduction of compatible, multivalent cationic fillers into cationic polymer matrices is an innovative approach to the design of flexible, structured membrane materials wherein the ionic moieties aid the selectivity and facilitation of CO_2_ through the film. The incorporation of ionic features through both the polymeric backbone and fillers/crosslinkers is explored in order to understand the enhancement in gas transport performance relative to the corresponding neat PIL, as well as demonstrate the potential for these PIL–IL composites as CO_2_ selective membranes for industrial separations from non-polar gases in flue gas, natural gas, or syngas. This work introduces the design, synthesis, and gas separation performance of a new series of di-, tri-, tetra- and hexa(imidazolium) benzene ILs in a photopolymerized polyelectrolyte matrix ([C_4_vim][Tf_2_N]). Additionally, the ([C_4_vim][Tf_2_N] matrix was also manipulated with extended ionic crosslinkers designed from tri- and tetra(imidazolium benzene) cores synthetically modified to possess reactive (vinyl imidazolium) groups at the periphery. The concept of such crosslinkers is a new approach in the design of PIL materials. The structural effects and transport behaviors of these composites were investigated, to gain an understanding of the structural–property relationships for these dispersed and crosslinked ionic composites and probe the influence of supplementary imidazolium moieties on the gas separation performance.

## 2. Materials and Methods

### 2.1. Materials

1,4-dibromobenzene (98%), potassium carbonate (99%, anhydrous), iodomethane (99%, stabilized), and 1,2,3,4,5,6-hexafluorobenzene (99%) were purchased from BeanTown Chemical (Hudson, NH, USA). 1,3,5-tribromobenzene (95%) was purchased from Matrix Scientific (Elgin, SC, USA). 1-bromobutane (98%) and 1,2,4,5-tetrabromobenzene were purchased from Alfa Aesar (Haverhill, MA, USA). 1-(3-aminopropyl) imidazole (API, 97%), 1-vinylimidazole (98%), sodium hydride (60% dispersion in Paraffin oil), and α,α’-Dichloro-p-xylene (>98%) were purchased from TCI (Tokyo, Japan). Dimethylformamide (DMF, anhydrous), toluene (anhydrous), Diethyl ether (Et_2_O, anhydrous), tetrahydrofuran (THF), acetonitrile (CH_3_CN), and N-methylpyrrolidone (NMP) were purchased from VWR (Radnor, PA, USA). LiTf_2_N was purchased from Iolitec (Heilbronn, Germany). Imidazole (99%) was purchased from Sigma Aldrich (St. Louis, MO, USA). Copper sulfate pentahydrate was purchased from BDH (Poole, UK). All pure gases (CO_2_, CH_4_, N_2_, O_2_—ultra high purity) for permeation testing were purchased from AirGas (Radnor, PA, USA). All materials were used as obtained, without further purification.

The synthetic procedure for the preparation of [C_4_vim][Tf_2_N] from 1-vinylimidazole and 1-bromobutane, followed by anion metathesis to the bistriflimide form, was performed as described in our prior works [33]. The vinyl IL was produced in our lab on a 100 g scale for use in these composite membranes.

### 2.2. Synthesis of Imidazolium Benzene Fillers and Crosslinker

#### 2.2.1. Synthesis of Di-, Tri-, Tetra- and Hexa(Imidazolium) Benzene ILs

The four imidazole-benzene derivatives have been reported in the literature, synthesized using similar coupling techniques [34,35,36]. 1,4-diimidazolebenzene, 1,3,5-triimidazolebenzene, and 1,2,4,5-tetraimidazolebenzene were prepared from the respective bromobenzene compounds via Ullmann couplings, with an excess of imidazole, K_2_CO_3_, and CuSO_4_·5H_2_O in NMP. The product was isolated by precipitation in deionized (DI) water and washed with 3 × 100 mL of DI water to remove residual solvent, base, and imidazole to yield the purified product. Hexaimidazolebenzene was prepared from hexafluorobenzene according to a literature procedure; however, this involved the deprotonation of imidazole in THF with NaH under an inert atmosphere, followed by the dropwise addition of hexafluorobenzene and DMF to yield the product.

**Scheme 1:**
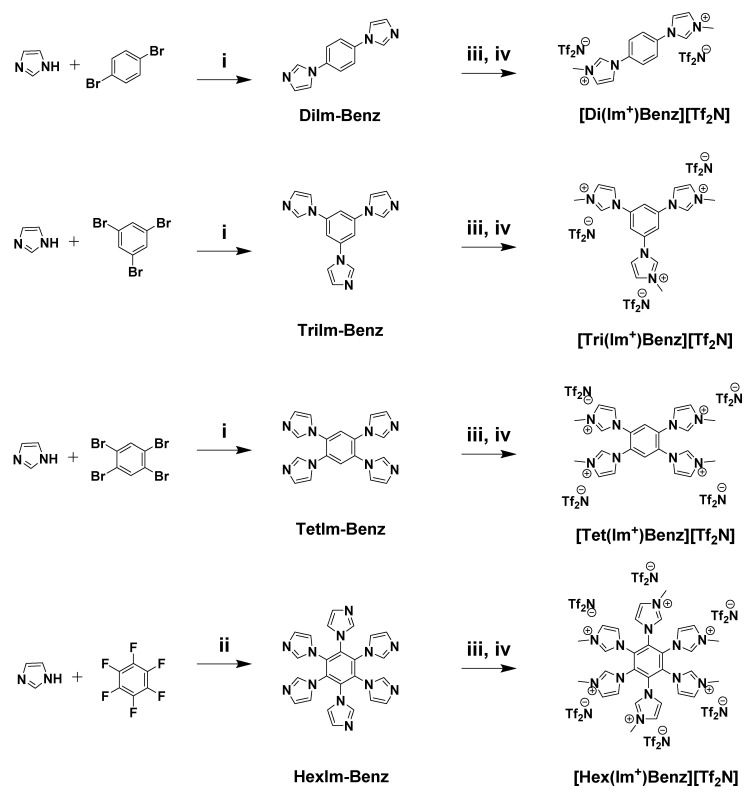
Imidazolium-benzene derived multivalent di-, tri-, tetra-, and hexa- imidazolium bistriflimide fillers. (i) K_2_CO_3_, CuSO_4_ ·5 H_2_O, NMP [150 °C, 24 h]; (ii) NaH, THF/DMF [65 °C, N_2_, 2 h]; (iii) MeI, DMF [80 °C, 16 h]; (iv) LiTf_2_N, H_2_O (RT, 2 h).

Subsequently, each imidazole-benzene derivative was quaternized to the methyl-imidazolium form using iodomethane, and then exchanged to the bistriflimide salt via precipitation in aqueous LiTf_2_N. The alkylation and ion exchange processes are detailed below (Scheme 1).

##### Synthesis of 1,1′-(1,4-Phenylene)bis(3-Methyl-1H-Imidazol-3-ium) Bistriflimide (“[Di(Im^+^)Benz][Tf_2_N]”)

1,4-di(1H-imidazol-1-yl)benzene (“DiIm-Benz”) (4.0 g, 19.0 mmol) was added with DMF (80 mL) to a 250 mL heavy-walled round-bottom pressure flask (Ace Glass, Vineland, NJ, USA) equipped with a stir bar. Iodomethane (10.8 g, 76 mmol) was carefully added, and the vessel was sealed with a PTFE screw cap with a Kalrez O-ring. The mixture was heated to 80 °C while stirring for 16 h, then was cooled to room temperature (RT). The solution was then poured into a solution of LiTf_2_N (24.0 g, 84 mmol) in deionized (DI) water and allowed to stir at RT for 2 h, to promote anion metathesis from the I^−^ salt and precipitate the product as the Tf_2_N^−^ salt. The precipitate was filtered and dried in an oven (Symphony Vacuum Oven, VWR, Radnor, PA, USA) at 100 °C overnight to yield the [Di(Im^+^)Benz ][Tf_2_N] product as a light tan crystalline solid (13.2 g, 87%). mp: 90–97 °C; ^1^H-NMR (d6-DMSO) [ppm] δ 9.87 (s, 2H), 8.40 (s, 2H), 8.10 (s, 4H), 8.01 (s, 2H), 3.99 (s, 6H).

##### Synthesis of 1,1′,1″-(Benzene-1,3,5-Triyl)tris(3-Methyl-1H-Imidazol-3-Ium) Bistriflimide (“[Tri(Im^+^)Benz][Tf_2_N]”)

“[Tri(Im+)Benz][Tf_2_N]” was prepared via a similar procedure. 1,3,5-tri(1H-imidazol-1-yl)benzene (“TriIm-Benz”) (4.0 g, 14.5 mmol) was added with DMF (80 mL) to a 250 mL heavy-walled round-bottom pressure flask (Ace Glass, Vineland, NJ, USA) equipped with a stir bar. Iodomethane (12.3 g, 87 mmol) was carefully added, the vessel was sealed, and the mixture was heated to 80 °C while stirring for 16 h. Upon cooling to RT, the solution was poured into aqueous LiTf_2_N (27.5 g, 96 mmol) solution and allowed to stir at RT for 2 h. The precipitate was filtered and dried in an oven at 100 °C overnight to yield the [Tri(Im^+^)Benz][Tf_2_N] product as an off-white powdery crystalline solid (14.7 g, 88%). mp: 107–110 °C; ^1^H-NMR (d6-DMSO) [ppm] δ 9.87 (s, 3H), 8.52 (s, 3H),8.40 (s, 3H), 8.11 (s, 3H), 4.07 (s, 9H).

##### Synthesis of 1,1′,1″,1′′′-(Benzene-1,2,4,5-Tetrayl)Tetrakis(3-Methyl-1H-Imidazol-3-Ium) Bistriflimide (“[Tet(Im^+^)Benz][Tf_2_N]”)

“[Tet(Im^+^)Benz][Tf_2_N]” was prepared via a similar procedure. 1,2,4,5-tetra(1H-imidazol-1-yl)benzene (“TetIm-Benz”) (4.0 g, 11.7 mmol) was added with DMF (80 mL) to a 250 mL heavy-walled round-bottom pressure flask (Ace Glass, Vineland, NJ, USA) equipped with a stir bar. Iodomethane (26.8 g, 93 mmol) was carefully added, the vessel was sealed, and the mixture was heated to 80 °C while stirring for 16 h. Upon cooling to RT, the solution was poured into aqueous LiTf_2_N (29.4 g, 102 mmol) solution and allowed to stir at RT for 2 h. The precipitate was filtered and dried in an oven at 100 °C overnight to yield the [Tet(Im^+^)Benz][Tf_2_N] product as a bright white powdery crystalline solid 17.2 g, 96%). mp: 216–219 °C; ^1^H-NMR (d6-DMSO) [ppm] δ 9.50 (s, 4H), 8.60 (s, 2H), 8.02 (s, 4H), 7.87 (s, 4H), 4.01 (s, 12H).

##### Synthesis of 1,1′,1″,1′′′,1′′′′,1′′′′′-(Benzene-1,2,3,4,5,6-Hexayl)Hexakis(3-Methyl-1H-Imidazol-3-Ium) Bistriflimide (“[Hexa(Im+)Benz][Tf_2_N]”)

“[Hexa(Im^+^)Benz][Tf_2_N]” was prepared via a similar procedure. 1,2,3,4,5,6-hexa(1H-imidazol-1-yl)benzene (“HexIm-Benz”) (4.0 g, 8.4 mmol) was added with DMF (80 mL) to a 250 mL heavy-walled round-bottom pressure flask (Ace Glass, Vineland, NJ, USA) equipped with a stir bar. Iodomethane (14.4 g, 101 mmol) was carefully added, the vessel was sealed, and the mixture was heated to 80 °C while stirring for 16 h. Upon cooling to RT, the solution was poured into aqueous LiTf_2_N (31.9 g, 111 mmol) solution and allowed to stir at RT for 2 h. The precipitate was filtered and dried under vacuum at 80 °C overnight. The product was recrystallized from acetone to yield the [Hexa(Im^+^)Benz][Tf_2_N] product as a brownish red soft crystalline solid (15.3 g, 81%). mp: 124–129 °C; ^1^H-NMR (d6-DMSO) (ppm) δ 9.02 (s, 6H), 7.64 (s, 12H), 3.86 (s, 18H).

#### 2.2.2. Synthesis of Imidazolium-Functionalized Tri- and Tetra Vinyl Crosslinking Agents

**Scheme 2:**
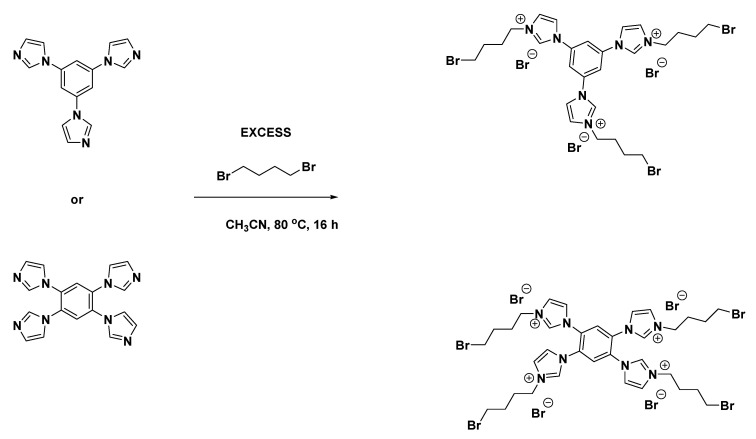
Imidazolium-benzene derived tri- or tetra- imidazolium butyl bromide precursors.

**Scheme 3:**
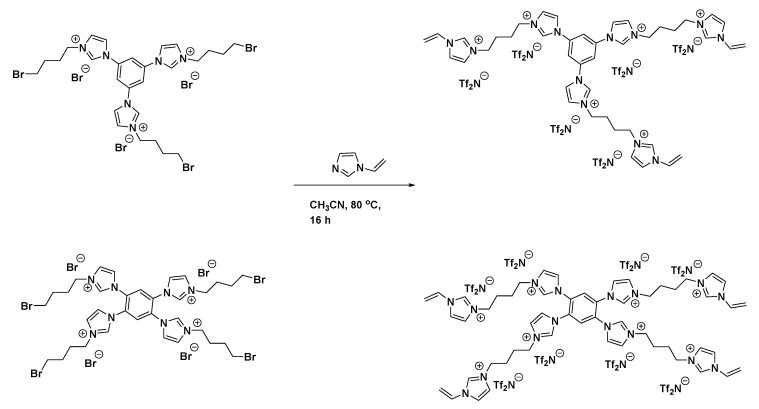
Imidazolium-benzene derived tri- or tetra- vinylimidazolium crosslinking agents.

Utilizing the same 1,3,5-triimidazolebenzene and 1,2,4,5-tetraimidazolebenzene precursors, a two-step synthetic process was utilized to produce ionic crosslinking agents with vinylimidazolium groups at the periphery (Scheme 2 and Scheme 3).

##### Synthesis of 1,1′,1″-(Benzene-1,3,5-Triyl)Tris(3-(4-(1-Vinyl-1H-Imidazol-3-Ium-3-yl)Butyl)-1H-Imidazol-3-Ium) Bistriflimide (“[Tri(VinylIm^+^)XL ][Tf_2_N]”)

1,3,5-tri(1H-imidazol-1-yl)benzene (“TriIm-Benz”) (4.0 g, 14.5 mmol) was added with CH_3_CN (120 mL) to a 250 mL heavy-walled round-bottom pressure flask (Ace Glass, Vineland, NJ, USA) equipped with a stir bar. 1,4-dibromodecane (18.8 g, 87 mmol) was carefully added while the mixture was stirring, the vessel was then sealed with a PTFE screw cap. The mixture was heated to 80 °C while stirring for 16 h. Upon cooling to RT, a gray gel precipitated. The solvent was decanted and disposed, and 200 mL of hexane was added and allowed to vigorously stir overnight, in order to remove excess 1,4-dibromobutane. This cloudy upper layer was decanted and disposed, and the 1,1′,1′′-(benzene-1,3,5-triyl)tris(3-(4-bromobutyl)-1H-imidazol-3-ium) bromide precursor was dried under vacuum at 40 °C then used directly in the next step. The precursor was dissolved in 100 mL of CH_3_CN and excess 1-vinylimidazole (5.45 g, 58 mmol) was added to the flask. The vessel was sealed and heated to 80 °C for 16 h. The reaction was cooled to room temperature, and was poured into aqueous LiTf_2_N (25.0 g, 87 mmol) then allowed to stir at RT for 2 h. The precipitate was filtered and dried under vacuum at 70 °C overnight to yield the Tri(VinylIm+)XL ][Tf_2_N] product isolated as a tan solid (21.6 g, 62%).^1^H NMR (360 MHz, DMSO) δ 9.47 (s, 3H), 8.23 (s, 6H), 8.15 (s, 3H), 8.00 (s, 6H), 7.93 (d, *J* = 1.6 Hz, 3H), 7.31 (d, *J* = 6.8 Hz, 3H), 5.99–5.94 (m, 3H), 5.46 (d, *J* = 9.0 Hz, 3H), 4.26 (s, 12H), 1.86 (s, 12H).

##### Synthesis of 1,1′,1″,1′′′-(Benzene-1,2,4,5-Tetrayl)Tetrakis(3-(4-(1-Vinyl-1H-Imidazol-3-Ium-3-yl)Butyl)-1H-Imidazol-3-Ium) Bistriflimide (“[Tet(VinylIm^+^)XL ][Tf_2_N]”)

Tet(VinylIm+)XL ][Tf_2_N] was prepared via a similar procedure. 1,2,4,5-tetra(1H-imidazol-1-yl)benzene (“TetIm-Benz”) (4.0 g, 11.7 mmol) was added with CH_3_CN (120 mL) to a 250 mL heavy-walled round-bottom pressure flask (Ace Glass, Vineland, NJ, USA) equipped with a stir bar. 1,4-dibromodecane (20.2 g, 93 mmol) was carefully added while the mixture was stirring, the vessel was then sealed with a PTFE screw cap. The mixture was heated to 80 °C while stirring for 16 h. Upon cooling to RT, a gray gel precipitated. The solvent was decanted and disposed, and 200 mL of hexane was added and allowed to vigorously stir overnight, in order to remove excess 1,4-dibromobutane. This cloudy upper layer was decanted and disposed, and the 1,1′,1′′,1′′′-(benzene-1,2,4,5-tetrayl)tetrakis(3-(4-bromobutyl)-1H-imidazol-3-ium) bromide precursor was dried under vacuum at 40 °C then used directly in the next step. The precursor was dissolved in 100 mL of CH_3_CN and excess 1-vinylimidazole (6.60 g, 70 mmol) was added to the flask. The vessel was sealed and heated to 80 °C for 16 h. The reaction was cooled to room temperature, and was poured into aqueous LiTf_2_N (26.9 g, 94 mmol) then allowed to stir at RT for 2 h. The precipitate was filtered and dried under vacuum at 70 °C overnight to yield the [Tet(VinylIm+)XL ][Tf_2_N] product isolated as a light brown solid (24.1 g, 65%). ^1^H NMR (360 MHz, DMSO) δ 9.45 (s, 4H), 8.23 (s, 4H), 8.10 (s, 4H), 7.92 (s, 4H), 7.90 (br, 2H), 7.33 (d, J = 8.1 Hz, 4H), 7.27 (br, 8H) 5.99–5.93 (m, 4H), 5.46 (dd, J = 8.6, 2.3 Hz, 4H), 4.25 (s, 16H), 1.85 (s, 16H).

### 2.3. Characterization

^1^H NMR data were obtained using 360 MHz or 500 MHz Bruker Avance instruments (Billerica, MA, USA). FTIR data were collected using a Perkin Elmer Spectrum Two ATR-FTIR instrument (Waltham, MA, USA). The wide-angle X-ray diffraction patterns of the materials were measured using a Bruker D8 Discover diffractometer (Billerica, MA, USA) by employing a scanning rate of 4° min^−1^ in a 2θ range from 17° to 70° with a Co Kα1 X-ray (λ = 0.17886 nm) source. The density of these composites was determined from triplicate experiments using a Mettler Toledo density kit and balance, which relies upon the Archimedes principle and the relative bulk sample weights in air and heptane (note that these composites are completely insoluble and do not interact with the PIL, fillers, or crosslinking agents). The d-spacing values were calculated using Bragg’s law (d = λ/(2sin θ)). Polarized light microscopy (PLM) images were obtained using a Leika DM 2700 P instrument (Wetzlar, Germany).

### 2.4. Composite and Membrane Fabrication

**Figure 2:**
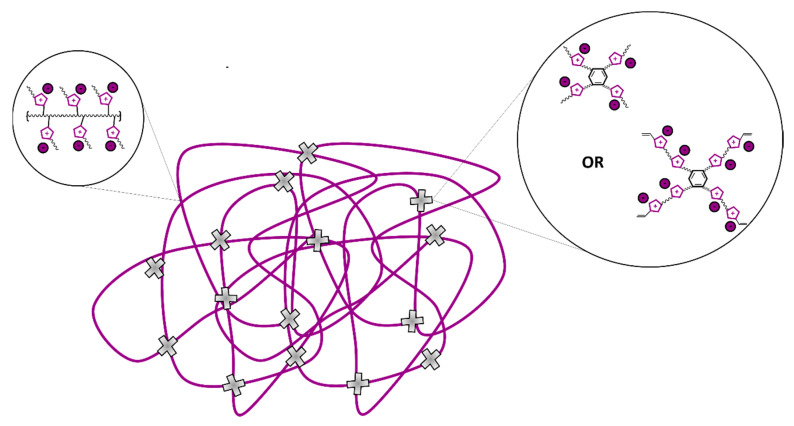
Visualization of poly(ILs) prepared from vinylimidazolium monomers polymerized with imidazolium-benzene based fillers or crosslinking agents in the membrane.

Composites with each of these imidazolium-benzene multivalent ILs or crosslinkers were prepared with consistent mass ratios, for comparison. Two different equivalencies of imidazolium-benzene multivalent vinyl imidazolium crosslinkers were evaluated, for comparison of the effects of the degree of crosslinking (Figure 2). Fresh vinyl IL monomer [C_4_vim][Tf_2_N] (0.9 g) was added to a glass scintillation vial with each imidazolium benzene filler (0.1 g) at room temperature (RT). For the crosslinked systems, 0.5 and 1.0 wt% mixtures were prepared with each crosslinker in [C_4_vim][Tf_2_N] at RT, due to the limited solubility and desired low degree of crosslinking. Then, a commercially available photoinitiator (2-hydroxy-2-methylpropiophenone) was added into each mixture at 0.5 wt% and each vial was agitated for 1 min to ensure the dissolution and dispersion of the additives. The resultant homogeneous solution was allowed to sit for 1 h to allow possible bubbles to settle and dissipate. The following photopolymerization methods have been successfully demonstrated previously by our group [37]. Each solution was then poured onto a clean Rain-X coated quartz plate and sandwiched by using a second identical quartz plate on the top. The plates were separated by punched circles of aluminum tape as edge spacers to control the membrane thickness. The plates were then irradiated under a 365 nm UV lamp (0.8 mW/cm^2^) for 24 h. Following the photopolymerization, the cured composite membrane was removed by the separation of the plates using a clean razor blade because of spacers, and the cross-linked anionic PIL-IL composite membrane was peeled off. The membrane thickness was controlled to be 122−237 μm, which were confirmed utilizing digital calipers. While the membrane thickness varied, all fell within a 115 μm range and in the same order of magnitude as the referenced neat material; further, the measured membrane thickness was taken into consideration for the gas transport calculations. These membranes were evaluated by additional characterization methods and pure gas permeation tests.

**Figure 3:**
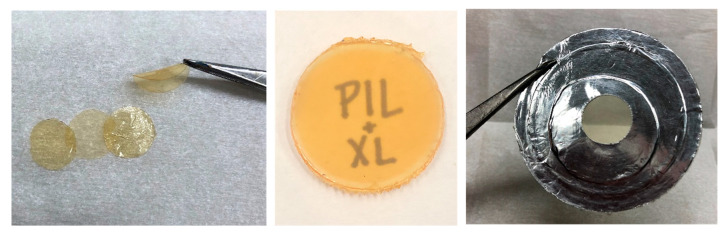
Transparency and flexibility of [C_4_vim][Tf_2_N] composites with imidazolium-benzene fillers (left); increased rigidity and opacity of [C_4_vim][Tf_2_N] composites with [Tri(VinylIm^+^)XL ][Tf_2_N] (right, as masked membrane) or [Tet (VinylIm^+^)XL ][Tf_2_N] (center, as punched disk).

The physical appearance and flexibility of these composite films are shown in Figure 3. It should be noted that the imidazolium-benzene-filled PILs are transparent and flexible, while the crosslinked derivatives are opaque and rigid.

## 3. Results and Discussion

### 3.1. Structural Characterizations

**Figure 4:**
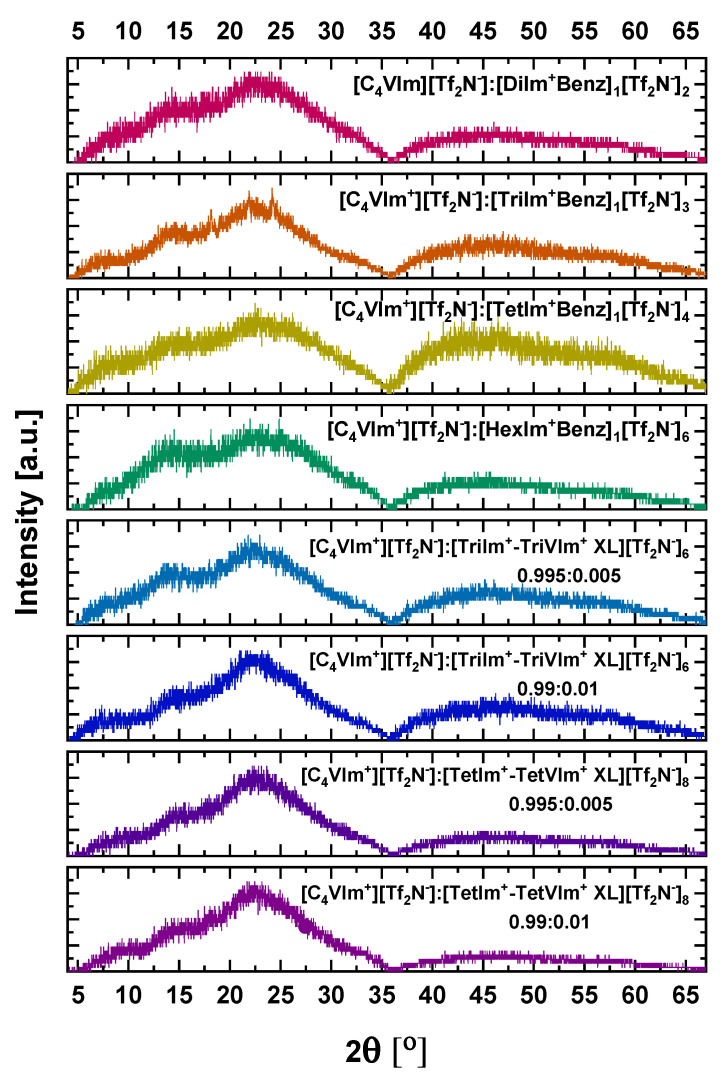
XRD profiles for all [C_4_vim][Tf_2_N] composite films.

XRD techniques were utilized to better understand the morphology of these composite films (Figure 4). A broad main halo was observed for all eight [C_4_vim][Tf_2_N] composites, with the center of the peak appearing within 2θ~21–25°. These maxima between 2θ~22–24° corresponds to an average d-spacing between 4.31 and 4.68 Å, representative of the interchain spacing between PIL chains. The dissolution and coordination of IL or XL initiates organization of domains within the photopolymerized ionic network. Multivalent IL introduce the apparent plateau adjacent to the main halo, in the 2θ~13–17°. This corresponds to d-spacings in the range of 6.05–7.89 Å. This XL derivatives exhibit a more pronounced peak in the 2θ~14.1–14.4° range, adjacent to the main halo. This corresponds to a d-spacing of approximately 7.25 Å. The improved permeabilities for derivatives 1–4 can be attributed to the influence of increased imidazolium content in each multivalent IL, enhancing the CO_2_ solubility within the composite films. For the crosslinked derivatives, however, we hypothesize that the CO_2_ permeability effects arise from both the increased ionic content as well as the structural influence of these crosslinking agents locking chains into more defined conformations and affecting entanglement.

**Figure 5:**
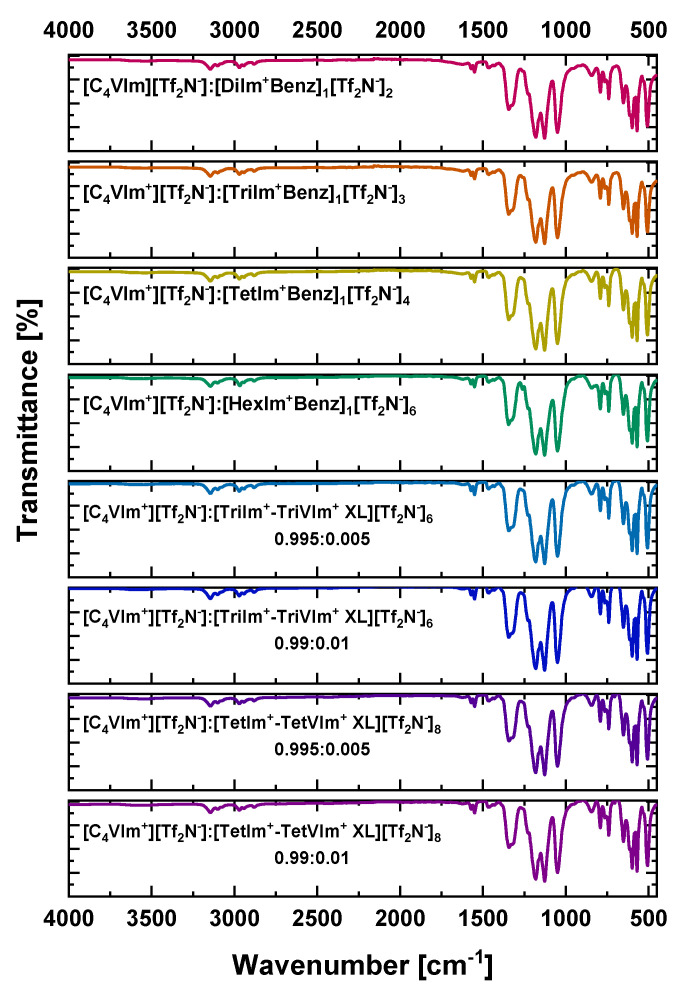
FTIR spectra for all [C_4_vim][Tf_2_N] composite films.

The chemical structure and functionality in each imidazolium-benzene filler or crosslinker was confirmed utilizing ^1^H-NMR (Supplementary Materials, Figures S1–S6). FTIR was also utilized (Figure 5) to ensure the presence of expected stretching and bending vibrations, which were observed for the expected imidazolium cation characteristic peaks (1021, 1337, 1378, 1570 cm^−1^), aromatic and alkyl (ν_C–H_ 2850–3150 cm^−1^), potentially residual weak vinyl/alkenyl (ν_C=C_ 1600 cm^−1^ & ν_C–H_ 3000–3150 cm^−1^) peaks, and bistriflimide anion characteristic peaks (1050, 1190, 1225, and 1348 cm^−1^) incorporated within these films [38].

Additional compositional and characterization details for these composite films are detailed below in Table 1. The mass and molar ratios for each vinyl IL + crosslinker or filler are summarized. The d-spacing values derived from primary peaks in the XRD analysis are included. Lastly, the bulk density of each composite film was tested, revealing that the tri- and hexa- imidazolium benzene-filled composites were the least dense, in comparison to the di- and tetra- imidazolium benzene-filled composites. This may be a result of enhanced stacking interactions contributed by the generally planar [Di(Im^+^)Benz ][Tf_2_N] and [Tet(Im^+^)Benz ][Tf_2_N] derivatives. The quantity of crosslinker added resulted in a subtle change in the density of the film, with slightly lower bulk densities observed for the films with 0.5 wt% crosslinker versus 1.0 wt% analogs.

**Figure 6:**
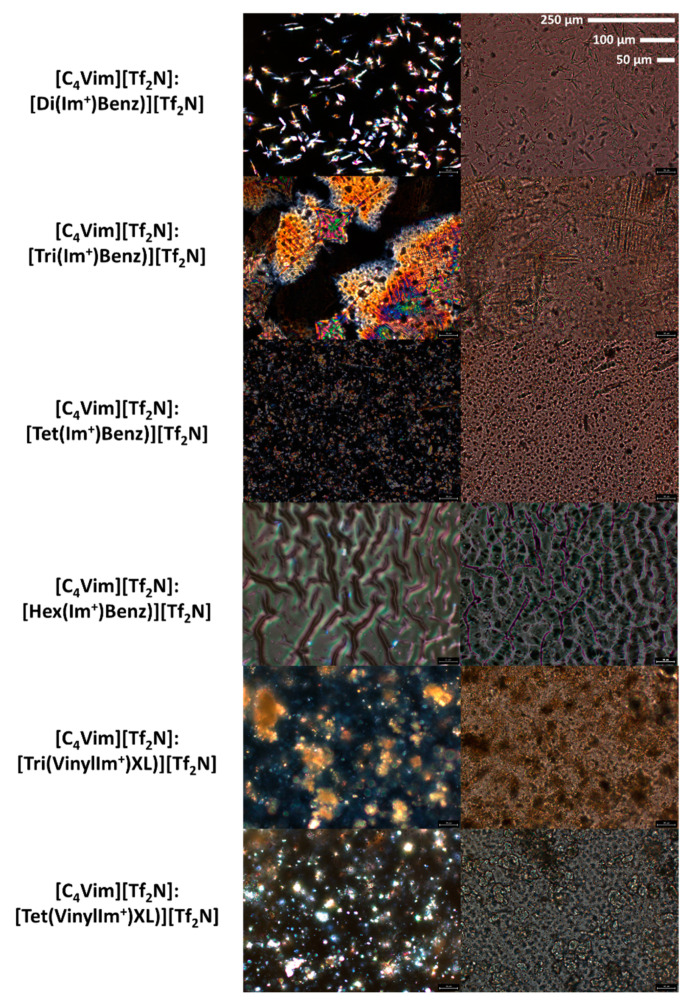
Polarized light microscopy (PLM) images of each thin film, at 20× magnification (reference enlarged scale bars), utilizing the polarized filter (left, 180°; right, 0°).

The homogeneity and morphology of these composite films was probed utilizing a polarized light microscope (PLM) at 20× magnification. While generally qualitative, the PLM images (Figure 6) demonstrate the pronounced effects of these crystalline imidazolium benzene fillers on the structuring within the PIL network. Under polarized light (180°), the highly ordered domains are visible. The regularity and distribution of these ordered domains are improved through the use of the vinylimidazolium crosslinkers, which may be a result of tethering PIL chains or a lower propensity to aggregate/force long range order in comparison to the “free” fillers.

### 3.2. Gas Transport Studies

The gas transport behaviors of these PIL: free IL or crosslinking IL composite films were investigated using a high-vacuum time-lag apparatus in our laboratories, based on the constant-volume/variable-pressure method, which have been detailed in our previous works [25,26,28,39,40]. To avoid membrane fracture due to edge pressure from the O-ring within the unit, the membranes were masked using an adhesive aluminum tape in order to confine gas permeation through a fixed membrane area of ½” diameter (A = 0.196 in^2^). All permeation measurements were conducted at 20 °C and the feed pressure was ~3 atm (~45 psia) against the initial downstream vacuum (<0.01 psia). Pressures and temperatures were monitored and recorded using LabVIEW software (National Instruments, Austin, TX, USA). Before each measurement, both the feed and the permeate sides were thoroughly evacuated to remove any residual gases. The permeate pressure increase versus time transient was recorded, and the permeability coefficient of each membrane was determined from the linear slope of the downstream pressure rise versus time plot (dp/dt). The ideal permselectivity (α_A/B_) for a pair of gases (A and B) was calculated from the ratio of the individual gas permeability coefficients as, P_A_/P_B_. For each gas feed (CO_2_, CH_4_, N_2_, and H_2_), experiments were performed in triplicate to derive the reported error. The corresponding permselectivities are included in Table 2.

**Figure 7:**
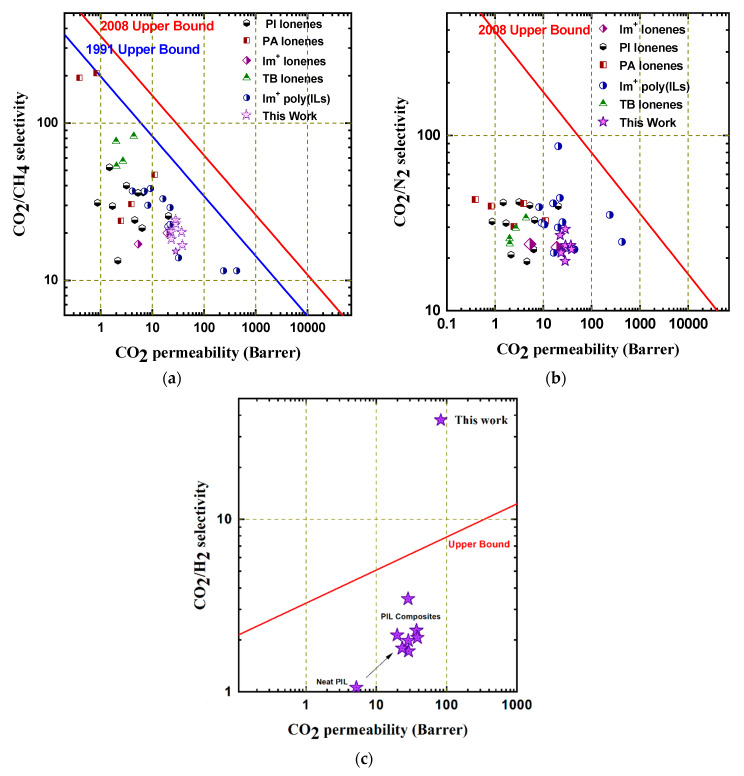
Robeson plots depicting the CO_2_ selectivity against N_2,_ CH_4,_ and H_2_ for the PIL:IL-filled membranes in this work for comparison with relevant membrane materials [4,26,28,37,39,40,41,43,44,45]. (**a**) α_CO2/CH4_ vs. P_CO2_; (**b**) α_CO2/N2_ vs. P_CO2;_ (**c**) α_CO2/H2_ vs. P_CO2._

These data highlight the significant improvement in the gas transport properties of [C_4_vim][Tf_2_N], when comparing the neat membrane with the PIL-IL composites. The permeability of CO_2_ for all derivatives exhibited a 4–7× increase, with the retention of selectivity or even an improvement of 80% for CO_2_/N_2._ It should also be noted that the P_H2_ also exhibited a 2–4× increase, which corresponded to a 2–3× increase in α_CO2/H2_ yet a decrease in α_H2/N2._ It should be highlighted that despite the small relative size of H_2_, these composites (like other PILs) all show selectivity for polar gases over non-polar, including P_CO2_ > P_H2_, due to interactions with CO_2_ and the membrane materials [42]. This relationship is significantly affected by the fillers or crosslinkers, as a clear shift toward the upper bound (increased P_CO2_ and P_CO2/H2_) was observed when comparing these composites (Figure 7c). The P_CO2_ was less affected by the “free” IL fillers, though even at these low concentrations there was a notable increase in P_CO2_ with the hexaimidazolium benzene filler, in comparison to the other derivatives. However, the highest selectivities were observed for composites containing the smallest di- and tris-imidazolium benzene fillers. A more pronounced effect was observed for the crosslinked composite films, which demonstrated the highest P_CO2_ for the tetra-vinylimidazolium crosslinking agent. While the P_CO2_ increased with increasing crosslinker, the permselectivities were marginally higher for composites with 0.5 wt% crosslinker in comparison with the 1.0 wt% derivatives. Generally, at the equivalent wt% of crosslinker, the P_CO2_ was higher for [Tet(VinylIm^+^)XL ][Tf_2_N] derivatives, whereas the selectivity was superior for composites with the [Tri(VinylIm^+^)XL ][Tf_2_N], specifically for gas pairs including CH_4_ and H_2_ (i.e., α_CO2/CH4_, α_H2/CH4_, α_H2/N2_).

The permeability is broken down into solubility and diffusivity contributions, which are calculated based on fundamental correlations (e.g., P_i_ = D_i_ × S_i_) and the time lag through the film according to the solution-diffusion model (Table 3) [12,39,41]. Diffusivity can be derived from the extrapolated slope of the steady state flux to the time axis, and solubility can be subsequently calculated from the pure gas permeability and diffusivity. While the variation in these composites is less pronounced, it is clear that the free imidazolium benzene additives affect the solubility of CO_2_ in the film. The solubility data reveal a consistent increase in the P_CO2_ with increasing imidazolium content in the filler (i.e., di < tri < tetra < hexa). The solubility and diffusivity of other gases remain relatively consistent, which is in agreement with the trends observed for CO_2_ selectivity, with CO_2_ solubility being the primary influence on CO_2_ permeability in these derivatives. While the CO_2_ solubility is generally higher, the relationship between crosslinker type and quantity with solubility and diffusivity data are less obvious, potentially due to the fact that these imidazolium functionalized crosslinkers participate dually as a functional and structural modifier of the PIL network.

The gas separation behaviors are highlighted below, which compare the permeability and selectivity of CO_2_/CH_4_, CO_2_/N_2,_ and CO_2_/H_2_ on Robeson plots relative to membrane materials with similar compositions (Figure 7). These composites demonstrate the ability to structurally modify [C_4_vim][Tf_2_N] via ionic fillers, in order to bring the CO_2_ permeability as well as CO_2_ selectivities (against CH_4_, N_2_, and H_2_) within the range of other leading, functionally similar ionic polymeric membranes. Relative to neat [C_4_vim][Tf_2_N], all of the composites included herein resulted in a shift toward the Robeson upper bound. While the enhanced gas transport properties for these composites relative to the corresponding neat PIL is primarily attributed to an increase in CO_2_ permeability, either based upon increased solubility (imidazolium-benzene fillers) or diffusivity (vinylimidazolium crosslinkers), it should be highlighted that the selectivity against non-polar gases is retained or even improved. These trends provide insight regarding the effects on each component on structuring the ionic matrix and how these features correlate to performance in membrane-based gas separations.

## 4. Conclusions

This study involved the preparation and analysis of a series of vinyl-imidazolium-based polyelectrolyte composites containing multivalent imidazolium-benzene ionic liquids (ILs) or novel cationic crosslinkers, reporting structural characterizations and gas transport performance. A set of eight [C_4_vim][Tf_2_N]-based membranes were probed to confirm functional features, and pure gas permeation data revealed the structural effects of “free” ILs dispersed in the polymeric matrix versus integrated ionic crosslinks, which were shown to impact the permeability of CO_2_ through these thin films.

These imidazolium PIL:IL composites exhibited moderately high CO_2_ permeabilities (~20–40 Barrer), with excellent selectivities against N_2_, CH_4_, and H_2._ The addition of imidazolium-benzene fillers with increased imidazolium content were shown to correspondingly enhance CO_2_ solubility (di- < tri- < tetra- < hexa-), with the [C_4_vim][Tf_2_N]:[Hex(Im^+^)Benz][Tf_2_N] composite showing the highest CO_2_ permeability (P_CO2_ = 38.4 Barrer), while maintaining modest selectivities (α_CO2/CH4_ = 20.2, α_CO2/N2_ = 23.6). Additionally, these metrics were similarly improved with the integration of more ionic content bonded to the polymeric matrix; increased P_CO2_ with increased wt% of the tri- and tetra-vinylimidazolium benzene crosslinking agent was observed. The permeability of CO_2_ for all derivatives exhibited a 4–7× increase and the permeability of H_2_ exhibited a 2–4X increase relative to neat [C_4_vim][Tf_2_N], with retention or enhanced selectivity for gas pairs including CO_2_/N_2_ or CO_2_/H_2._ This study demonstrates the intriguing interactions and effects of ionic additives or crosslinkers within a PIL matrix, revealing the potential for the tuning the properties and gas transport behaviors of ionic polymers using ionic liquid=inspired small molecules. Specifically, this work illustrates the suitability and tailorability of PIL composites with added ionic fillers, which impart order and enhance the permeability and selectivity in these gas separation membranes. Future work expanding upon these concepts will involve further modification of the structure, concentration, and nature of charged features of these imidazolium-benzene-based additives, in order to explore the structure–property relationships and separation performance of other polyelectrolytes, ionenes, or other ionic macromolecular architectures.

## Figures and Tables

**Table 1:** Composition and characterization data for each PIL composite.

ID	Ionene Composite	Mass Ratio	Molar Ratio	d-Spacing (Å)	Density (g/cm^3^)	MWadd (g/mol)	MWPIL (g/mol)
1	[C4vim][Tf2N]: [Di(Im^+^)Benz ][Tf2N]	0.9:0.1	0.944:0.056	4.485	7.099	1.569	431.37
2	[C4vim][Tf2N]: [Tri(Im^+^)Benz ][Tf2N]	0.9:0.1	0.960:0.040	4.567	7.079	1.462	
3	[C4vim][Tf2N]: [Tet(Im^+^)Benz ][Tf2N]	0.9:0.1	0.969:0.031	4.380	7.338	1.562	
4	[C4vim][Tf2N]: [Hex(Im^+^)Benz ][Tf2N]	0.9:0.1	0.979:0.021	4.392	7.433	1.377	
5	[C4vim][Tf2N]: [Tri(VinylIm^+^)XL ][Tf2N]	0.99:0.01	0.983:0.017	4.585	6.904	1.528	
6	[C4vim][Tf2N]: [Tet(VinylIm^+^)XL ][Tf2N]	0.99:0.01	0.987:0.013	4.576	6.904	1.480	
7	[C4vim][Tf2N]: [Tri(VinylIm^+^)XL ][Tf2N]	0.995:0.005	0.991:0.009	4.568	7.359	1.461	
8	[C4vim][Tf2N]: [Tet(VinylIm^+^)XL ][Tf2N]	0.995:0.005	0.993:0.007	4.521	6.904	1.463	

**Table 2:** Pure gas permeability and selectivity data for each PIL composite.

ID	Ionene Composite	Mass Ratio	Data ^a^							
Permeability and Selectivity			P_CO2_	±	P_N2_	±	P_CH4_	±	P_H2_	±
*	[C_4_vim][Tf_2_N] Neat	1.0:0.0	5.2	-	0.3	-	-	-	4.9	-
1	[C_4_vim][Tf_2_N]: [Di(Im^+^)Benz ][Tf_2_N]	0.9:0.1	28.7	0.19	1.22	0.05	1.26	0.05	16.6	0.15
2	[C_4_vim][Tf_2_N]: [Tri(Im^+^)Benz ][Tf_2_N]	0.9:0.1	28.5	1.11	0.98	0.03	1.18	0.09	14.4	0.16
3	[C_4_vim][Tf_2_N]: [Tet(Im^+^)Benz ][Tf_2_N]	0.9:0.1	28.3	0.44	1.47	0.04	1.85	0.17	8.16	0.05
4	[C_4_vim][Tf_2_N]: [Hex(Im^+^)Benz ][Tf_2_N]	0.9:0.1	37.2	0.83	1.58	0.05	1.84	0.10	16.4	0.08
5	[C_4_vim][Tf_2_N]: [Tri(VinylIm^+^)XL ][Tf_2_N]	0.99:0.01	23.5	0.50	1.08	0.04	1.29	0.08	13.2	0.03
6	[C_4_vim][Tf_2_N]: [Tet(VinylIm^+^)XL ][Tf_2_N]	0.99:0.01	38.4	0.42	1.72	0.04	2.31	0.09	18.7	0.10
7	[C_4_vim][Tf_2_N]: [Tri(VinylIm^+^)XL ][Tf_2_N]	0.995:0.005	19.8	0.38	0.63	0.01	0.81	0.03	9.29	0.55
8	[C_4_vim][Tf_2_N]: [Tet(VinylIm^+^)XL ][Tf_2_N]	0.995:0.005	23.3	0.40	1.09	0.05	1.15	0.05	13.0	0.04
**Permeability and Selectivity**			**α_CO2/N2_**	**α_CO2/CH4_**	**α_CO2/H2_**	**α_H2/CH4_**	**α_H2/N2_**	**Film Thickness (μm)**		
*	[C_4_vim][Tf_2_N] Neat	1.0:0.0	17.3	-	1.06	-	16.3	150		
1	[C_4_vim][Tf_2_N]: [Di(Im^+^)Benz ][Tf_2_N]	0.9:0.1	23.6	22.8	1.72	13.21	13.7	130		
2	[C_4_vim][Tf_2_N]: [Tri(Im^+^)Benz ][Tf_2_N]	0.9:0.1	29.2	24.1	1.98	12.12	14.7	130		
3	[C_4_vim][Tf_2_N]: [Tet(Im^+^)Benz ][Tf_2_N]	0.9:0.1	19.2	15.3	3.46	4.42	5.55	237		
4	[C_4_vim][Tf_2_N]: [Hex(Im^+^)Benz ][Tf_2_N]	0.9:0.1	23.6	20.2	2.27	8.91	10.4	155		
5	[C_4_vim][Tf_2_N]: [Tri(VinylIm^+^)XL ][Tf_2_N]	0.99:0.01	21.8	18.2	1.79	10.17	12.2	122		
6	[C_4_vim][Tf_2_N]: [Tet(VinylIm^+^)XL ][Tf_2_N]	0.99:0.01	22.4	16.6	2.06	8.07	10.8	190		
7	[C_4_vim][Tf_2_N]: [Tri(VinylIm^+^)XL ][Tf_2_N]	0.995:0.005	31.5	24.6	2.13	11.53	14.8	148		
8	[C_4_vim][Tf_2_N]: [Tet(VinylIm^+^)XL ][Tf_2_N]	0.995:0.005	21.4	20.2	1.79	11.30	12.0	142		

^a^ Pure gas permeability (P_i_) reported in Barrer, * neat membrane data as reported from reference [41].

**Table 3:** Pure gas diffusivity and solubility data for each PIL composite.

ID	Ionene Composite	Mass Ratio	Data ^a^							
Diffusivity and Solubility			D_CO2_	±	D_N2_	±	D_CH4_	±	D_H2_	±
1	[C_4_vim][Tf_2_N]: [Di(Im^+^)Benz ][Tf_2_N]	0.9:0.1	1.10 × 10^−7^	6.53 × 10^−9^	5.86 × 10^−7^	5.53 × 10^−7^	7.10 × 10^−8^	5.00 × 10^−9^	5.41 × 10^−7^	9.23 × 10^−8^
2	[C_4_vim][Tf_2_N]: [Tri(Im^+^)Benz ][Tf_2_N]	0.9:0.1	1.00 × 10^−7^	1.54 × 10^−8^	5.78 × 10^−8^	2.34 × 10^−8^	3.46 × 10^−8^	7.25 × 10^−9^	2.61 × 10^−7^	9.99 × 10^−8^
3	[C_4_vim][Tf_2_N]: [Tet(Im^+^)Benz ][Tf_2_N]	0.9:0.1	9.53 × 10^−8^	1.13 × 10^−9^	2.69 × 10^−8^	1.09 × 10^−9^	2.61 × 10^−8^	3.36 × 10^−9^	3.53 × 10^−7^	9.70 × 10^−8^
4	[C_4_vim][Tf_2_N]: [Hex(Im^+^)Benz ][Tf_2_N]	0.9:0.1	9.44 × 10^−8^	6.58 × 10^−9^	2.39 × 10^−8^	4.48 × 10^−9^	2.55 × 10^−8^	8.43 × 10^−9^	2.24 × 10^−7^	5.57 × 10^−8^
5	[C_4_vim][Tf_2_N]: [Tri (VinylIm^+^)XL ][Tf_2_N]	0.99:0.01	6.66 × 10^−8^	4.29 × 10^−9^	1.63 × 10^−8^	8.88 × 10^−10^	9.59 × 10^−9^	1.67 × 10^−9^	4.75 × 10^−7^	8.12 × 10^−8^
6	[C_4_vim][Tf_2_N]: [Tet (VinylIm^+^)XL ][Tf_2_N]	0.99:0.01	2.45 × 10^−7^	3.78 × 10^−8^	3.91 × 10^−8^	5.78 × 10^−9^	1.41 × 10^−7^	2.20 × 10^−8^	1.81 × 10^−7^	4.33 × 10^−8^
7	[C_4_vim][Tf_2_N]: [Tri (VinylIm^+^)XL ][Tf_2_N]	0.995:0.005	7.99 × 10^−8^	4.51 × 10^−9^	6.02 × 10^−7^	8.83 × 10^−8^	1.87 × 10^−7^	1.86 × 10^−8^	1.2 × 10^−7^	5.00 × 10^−10^
8	[C_4_vim][Tf_2_N]: [Tet (VinylIm^+^)XL ][Tf_2_N]	0.995:0.005	4.57 × 10^−8^	1.01 × 10^−8^	1.86 × 10^−8^	7.71 × 10^−9^	2.47 × 10^−8^	3.73 × 10^−9^	2.59 × 10^−7^	6.14 × 10^−8^
**Diffusivity and Solubility**			**S_CO2_**	**±**	**S_N2_**	**±**	**S_CH4_**	**±**	**S_H2_**	**±**
1	[C_4_vim][Tf_2_N]: [Di(Im^+^)Benz ][Tf_2_N]	0.9:0.1	1.98	0.122	0.15	0.139	0.14	0.010	0.24	0.039
2	[C_4_vim][Tf_2_N]: [Tri(Im^+^)Benz ][Tf_2_N]	0.9:0.1	2.19	0.239	0.16	0.068	0.29	0.076	0.46	0.157
3	[C_4_vim][Tf_2_N]: [Tet(Im^+^)Benz ][Tf_2_N]	0.9:0.1	2.25	0.062	0.42	0.004	0.54	0.009	0.35	0.120
4	[C_4_vim][Tf_2_N]: [Hex(Im^+^)Benz ][Tf_2_N]	0.9:0.1	3.01	0.274	0.59	0.102	0.46	0.087	0.59	0.129
5	[C_4_vim][Tf_2_N]: [Tri (VinylIm^+^)XL ][Tf_2_N]	0.99:0.01	3.53	0.230	0.51	0.008	1.02	0.008	0.21	0.090
6	[C_4_vim][Tf_2_N]: [Tet (VinylIm^+^)XL ][Tf_2_N]	0.99:0.01	1.56	0.157	0.33	0.012	0.12	0.062	0.78	0.142
7	[C_4_vim][Tf_2_N]: [Tri (VinylIm^+^)XL ][Tf_2_N]	0.995:0.005	2.47	0.872	0.02	0.006	0.03	0.006	0.61	0.005
8	[C_4_vim][Tf_2_N]: [Tet (VinylIm^+^)XL ][Tf_2_N]	0.995:0.005	5.09	0.967	0.54	0.244	0.36	0.071	0.40	0.095

^a^ Diffusivity coefficient (D) [cm^2^/s], solubility coefficient (S) [10^−2^ cm_STP_^3^ cm^−3^ cm Hg^−1^].

## Supplementary Materials

The following are available online at https://www.mdpi.com/article/10.3390/polym13091388/s1, Supplementary Figures S1–S6: NMR Spectra, Supplementary Figure S7: XRD profiles for recrystallized fillers.
