# Supplementary material for: Design and Gas Separation Performance of Imidazolium Poly(ILs) Containing Multivalent Imidazolium Fillers and Crosslinking Agents
Source: Polymers (Basel). 2021 Apr 24;13(9):1388. doi: 10.3390/polym13091388 (PMC8123196; doi:10.3390/polym13091388)
Supplement: Supplementary file 1 [file polymers-13-01388-s001.zip › polymers-1194953-supplementary.pdf]

polymers

MDPI

SUPPORTING INFORMATION

for

Design and Gas Separation Performance of Imidazolium Poly(ILs) Containing Multivalent Imidazolium Fillers and Crosslinking Agents

Kathryn E. O'Harra, Emily DeVriese, Erika Turflinger, Danielle Noll, and Jason E. Bara

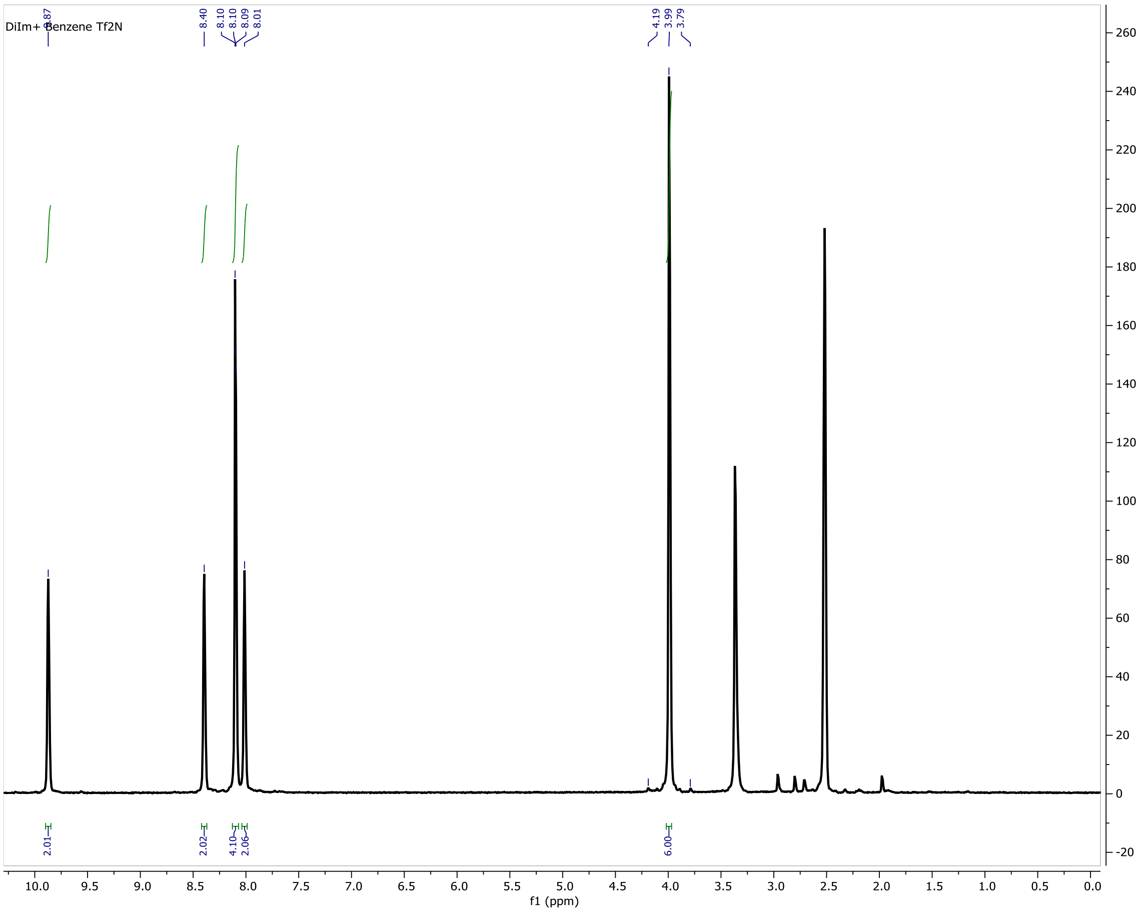

^{1}H-NMR (d6-DMSO) [ppm] δ 9.87 (s, 2H), 8.40 (s, 2H), 8.10 (s, 4H), 8.01 (s, 2H), 3.99 (s, 6H).

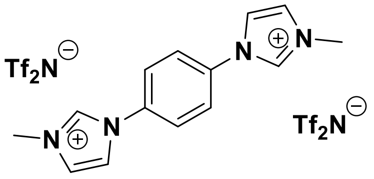

Figure S1: ^{1}H-NMR spectrum for [Di(Im+)Benz)][Tf_2N].

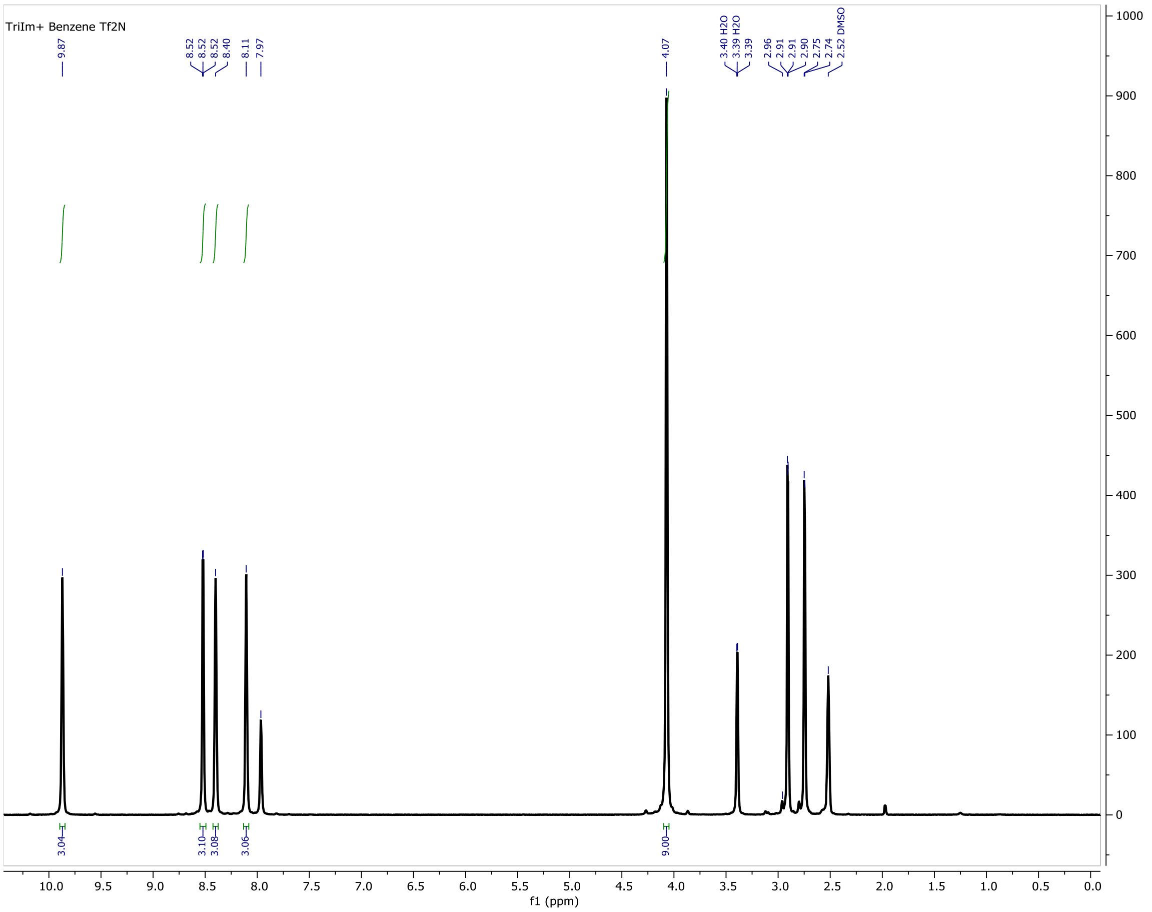

^{1}H-NMR (d6-DMSO) [ppm] δ 9.87 (s, 3H), 8.52 (s, 3H), 8.40 (s, 3H), 8.11 (s, 3H), 4.07 (s, 9H).

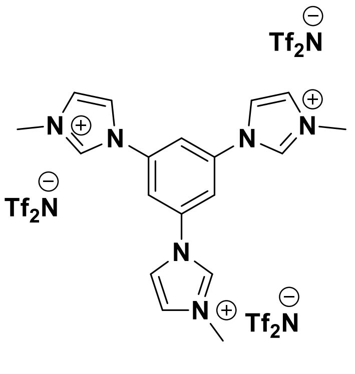

(some residual DMF was observed)

Figure S2: ^{1}H-NMR spectrum for [Tri(Im+)Benz)][Tf_2N].

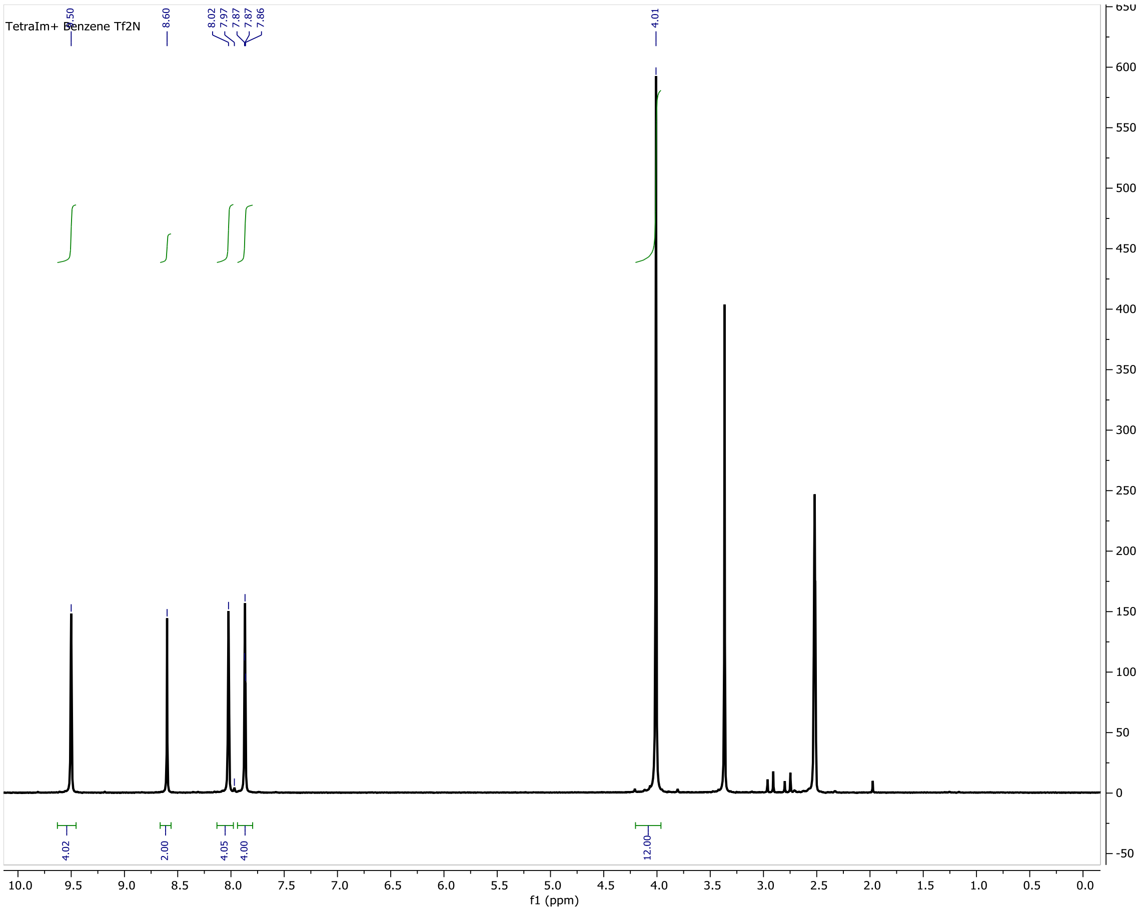

^{1}H-NMR (d6-DMSO) [ppm] δ 9.50 (s, 4H), 8.60 (s, 2H), 8.02 (s, 4H), 7.87 (s, 4H), 4.01 (s, 12H).

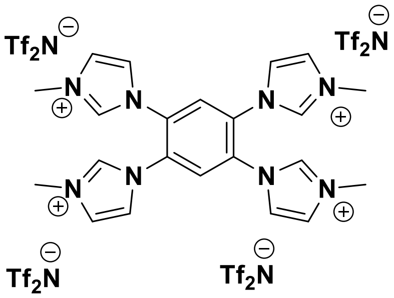

Figure S3: ^{1}H-NMR spectrum for [Tetra(Im+)Benz)][Tf_2N].

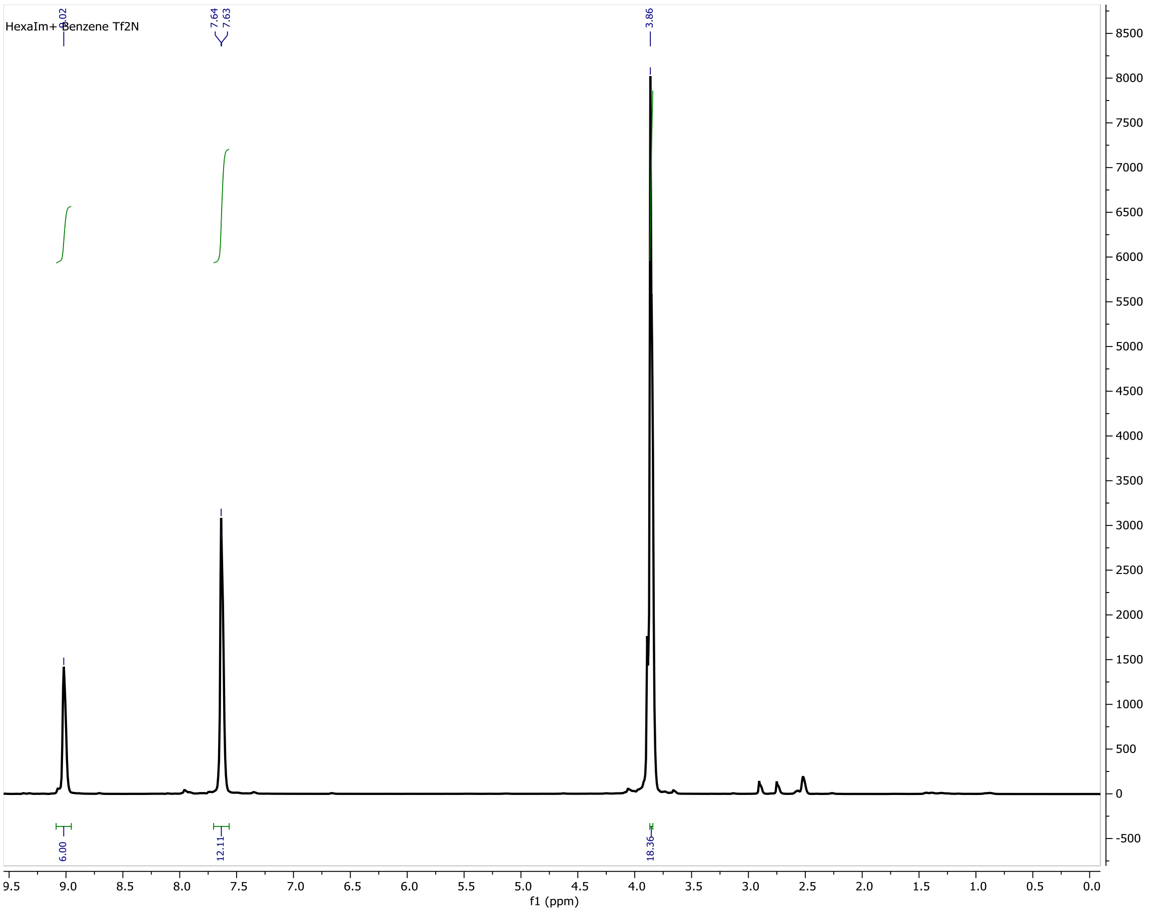

¹H-NMR (d6-DMSO) [ppm] δ 9.02 (s, 6H), 7.64 (s, 12H), 3.86 (s, 18H).

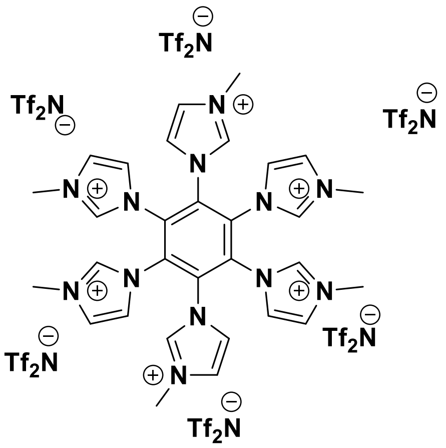

Figure S4: ¹H-NMR spectrum for [Hexa(Im+)Benz)][Tf₂N].

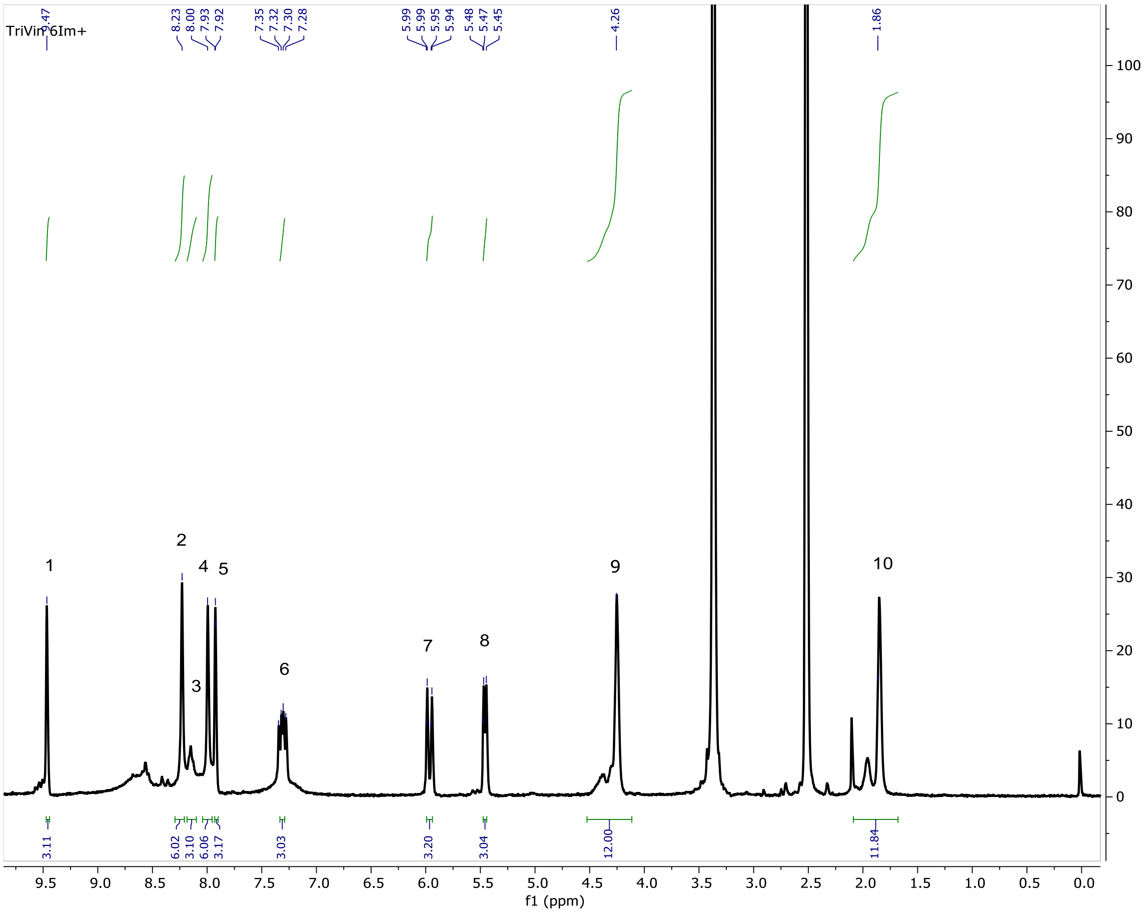

^{1}H NMR (360 MHz, DMSO) δ 9.47 (s, 3H), 8.23 (s, 6H), 8.15 (s, 3H), 8.00 (s, 6H), 7.93 (d, *J* = 1.6 Hz, 3H), 7.31 (d, *J* = 6.8 Hz, 3H), 5.99 – 5.94 (m, 3H), 5.46 (d, *J* = 9.0 Hz, 3H), 4.26 (s, 12H), 1.86 (s, 12H).

Figure S5: ^{1}H-NMR spectrum for [Tri(VinylIm+)XL)][Tf_2N].

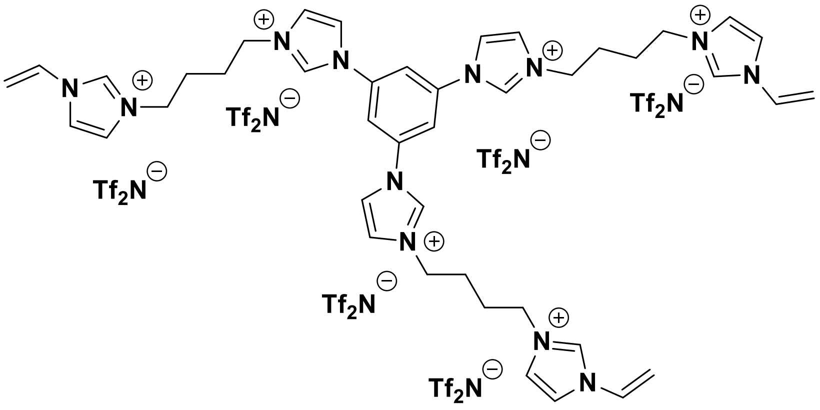

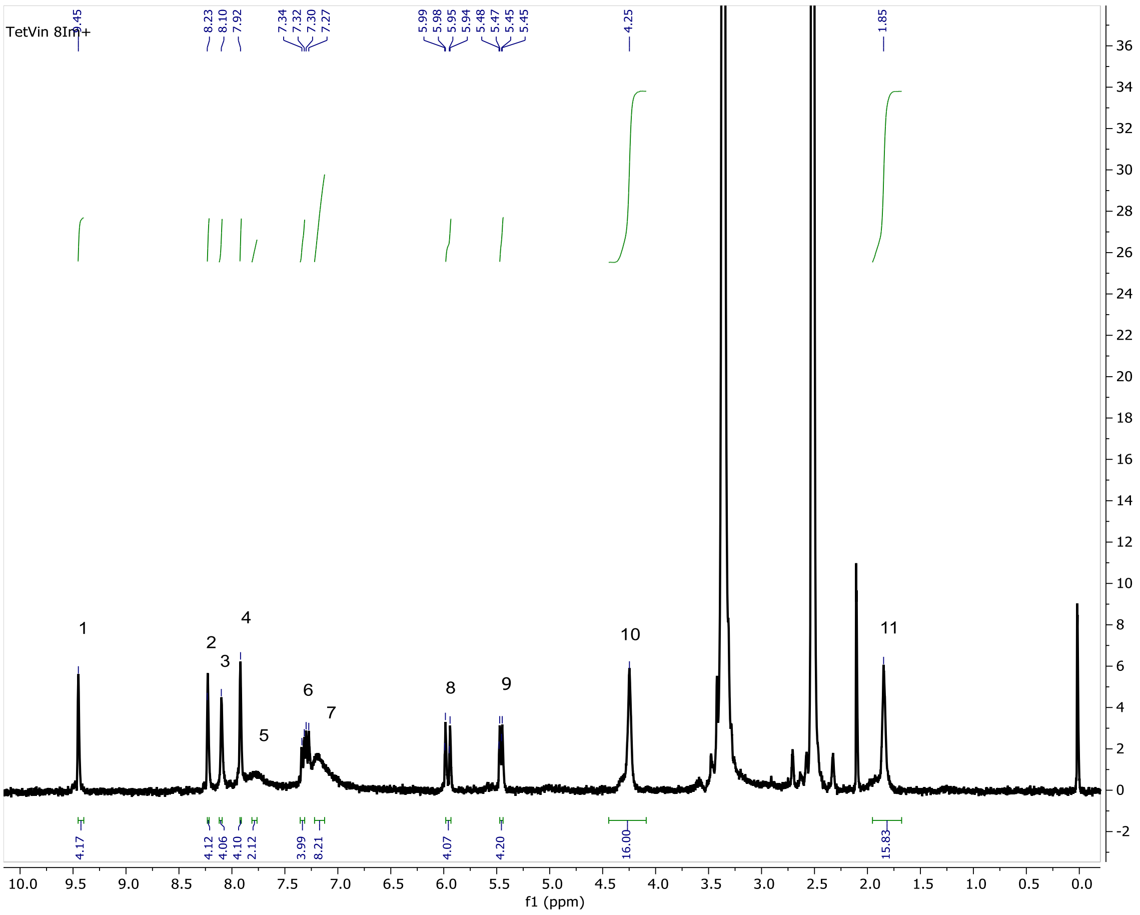

^{1}H NMR (360 MHz, DMSO) δ 9.45 (s, 4H), 8.23 (s, 4H), 8.10 (s, 4H), 7.92 (s, 4H), 7.90 (br, 2H), 7.33 (d, *J* = 8.1 Hz, 4H), 7.27 (br, 8H) 5.99 – 5.93 (m, 4H), 5.46 (dd, *J* = 8.6, 2.3 Hz, 4H), 4.25 (s, 16H), 1.85 (s, 16H).

Figure S6: ^{1}H-NMR spectrum for [Tet(VinylIm+)XL)][Tf_2N].

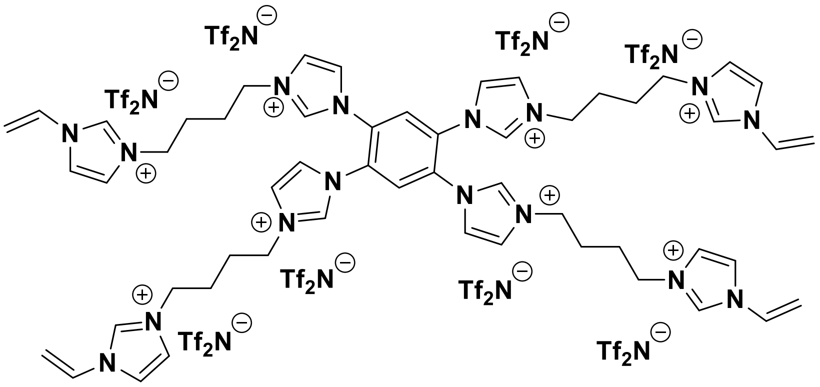

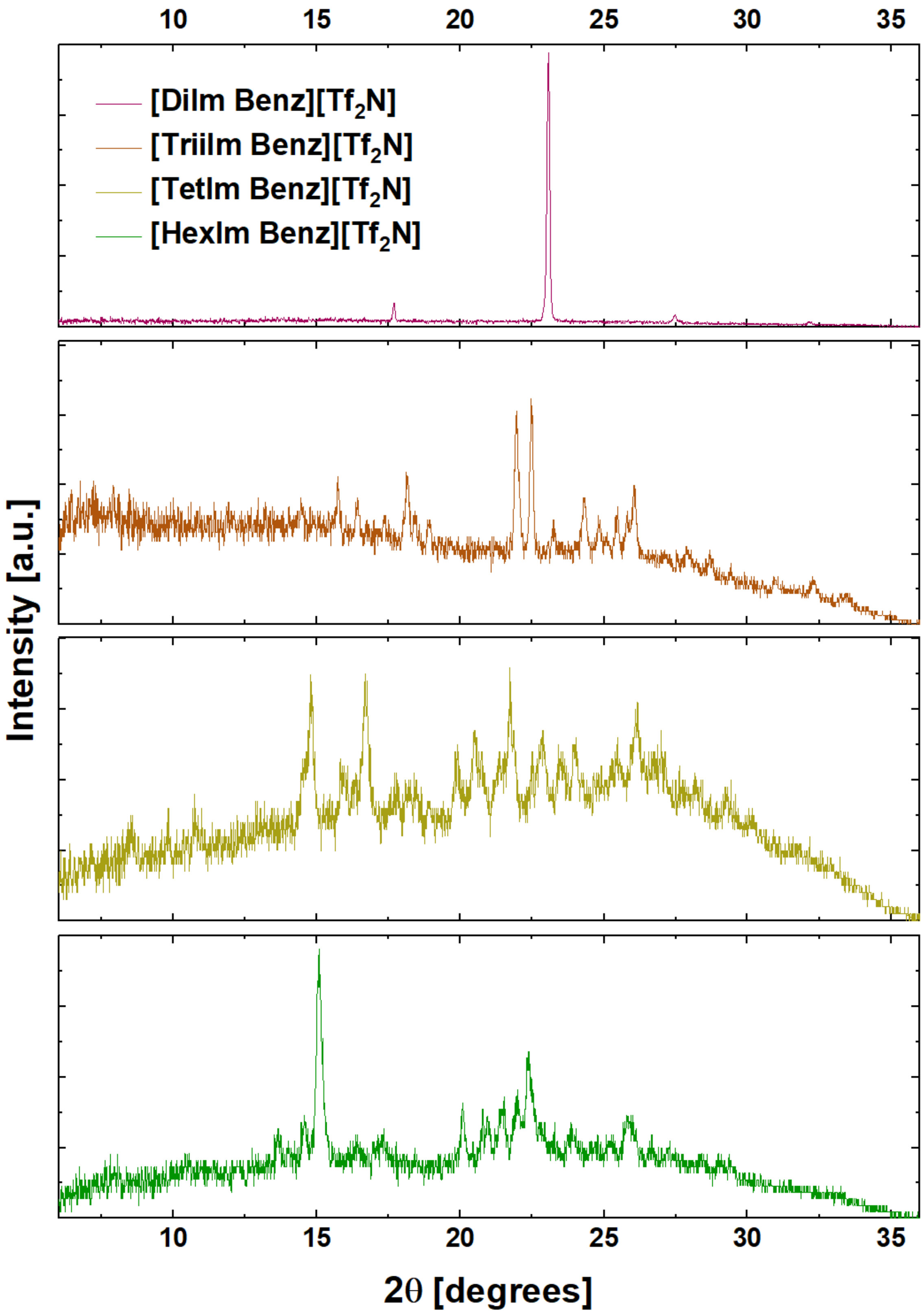

Figure S7: XRD profiles for each recrystallized multivalent filler.
